# Dynamic Statistical Mechanics Modeling of Percolation Networks in Conductive Polymer Composites for Smart Sensor Applications

**DOI:** 10.3390/ma18133097

**Published:** 2025-06-30

**Authors:** Sang-Un Kim, Joo-Yong Kim

**Affiliations:** 1 Department of Smart Wearable Engineering, Soongsil University, Seoul 06978, Republic of Korea; tkddnsl0723@naver.com; 2 Department of Materials Science and Engineering, Soongsil University, Seoul 06978, Republic of Korea

**Keywords:** conductive polymer composites (CPCs), dynamic statistical mechanics, Monte Carlo simulation, percolation theory

## Abstract

Conductive polymer composites (CPCs) are widely used in flexible electronics due to their tunable electrical properties and mechanical deformability. However, accurately predicting the evolution of conductive networks, particularly under compressive strain, remains a significant challenge. In this study, we developed a statistical mechanics model and an extended dynamic statistical mechanics model to quantitatively describe percolation behavior in CPCs. The static model incorporates filler geometry, aspect ratio (AR), and surface-to-volume ratio, and was validated using Monte Carlo simulations. Results show that the percolation threshold for spherical fillers was 0.11965, while significantly lower values of 0.00669 and 0.00203 were observed for plate- and rod-shaped fillers, respectively, confirming the enhanced connectivity of anisotropic particles. To capture strain-dependent behavior, a dynamic model was constructed using a Smoluchowski-type gain–loss framework. This model separates conductive network formation (gain) from network disconnection (loss) caused by filler alignment and Poisson-induced expansion. At high Poisson’s ratios (0.3 and 0.5), the model accurately predicted the reduction in connectivity, particularly for anisotropic fillers. Across all tested conditions, the model exhibited strong agreement with simulation data, with RMSE values ranging from 0.0004 to 0.0449. The results confirm that high AR fillers enhance conductivity under compression, while large Poisson’s ratios suppress network formation. These findings provide a reliable, physically grounded modeling framework for designing strain-sensitive devices such as flexible pressure sensors.

## 1. Introduction

Conductive polymer composites (CPCs) are widely researched due to their promising potential in flexible electronics, particularly in wearable pressure sensors [1,2,3,4]. These materials combine the mechanical flexibility of polymer matrices with the electrical conductivity provided by embedded conductive fillers [5,6,7]. Commonly used polymer matrices in CPCs include polyvinyl alcohol (PVA) [8], Ecoflex [9,10], silicone [11,12], and rubber [13], chosen for their flexibility, stretchability, and ease of processing. To impart electrical conductivity, various types of conductive fillers are incorporated into these matrices, such as carbon black [14,15,16], carbon nanotubes (CNTs) [6,17,18,19,20], and emerging plate-like materials like MXenes [21,22,23]. The combination of these matrix–filler systems allows CPCs to exhibit tunable electromechanical responses suitable for strain or pressure sensing applications.

The electrical behavior of CPCs is governed by the formation of percolated conductive networks, a phenomenon commonly described by classical percolation theory [24,25]. According to this theory, when the volume fraction of conductive fillers exceeds a critical threshold, a continuous conductive path spanning the material emerges, drastically enhancing its conductivity [26,27].

However, conventional percolation models often overlook critical factors inherent to real CPC systems, such as the filler’s geometric anisotropy [28,29,30], surface-to-volume ratio [31,32], and spatial orientation [33,34]. These parameters significantly influence the network formation probability and the effective percolation threshold. Moreover, in practical applications such as wearable sensors, CPCs are frequently subjected to mechanical deformation, particularly compressive strain, which dynamically alters the internal structure of the material [17,35]. Compressive strain can reduce inter-particle distances and promote connectivity [36,37], but it can simultaneously induce filler alignment and lateral expansion due to the Poisson effect, potentially disrupting existing conductive pathways [24,37,38].

In recent years, Monte Carlo simulation has emerged as a powerful tool to analyze the formation of conductive networks and predict percolation thresholds in composite materials. For example, Zhang et al. [39] employed a particle-based Monte Carlo model to investigate the effective conductivity of geopolymer–graphite composites, showing how pore size distribution and filler polydispersity influence network connectivity. Similarly, Xing et al. [40] applied Monte Carlo finite-size scaling to study the shape-dependent percolation threshold in tetrahedron-to-sphere evolving particles. These studies highlight the relevance of Monte Carlo methods for capturing the statistical nature of percolation phenomena in both undeformed and deformed states.

To accurately describe these competing phenomena of network formation and disconnection under strain, we propose a dynamic statistical mechanics model. This model extends a foundational statistical mechanics model, which incorporates filler shape (spherical, rod-like, or plate-like), aspect ratio (AR), and alignment into classical percolation theory to estimate static connectivity. The dynamic extension introduces a gain–loss formalism inspired by Marian Smoluchowski’s coagulation equation [41], which originally described time-dependent aggregation in colloidal systems. In this framework, the gain term reflects the formation of conductive networks via densification of fillers under compressive strain, while the loss term captures the network disruption caused by filler reorientation and lateral expansion due to the Poisson effect.

Critically, unlike conventional static models that predict connectivity based on fixed states, the proposed model dynamically tracks how conductive pathways evolve as a function of external compressive strain. This allows the model to describe not only the threshold behavior, but also the nonlinear and temporally evolving connectivity of the conductive network during deformation. The model thus offers a more physically grounded and predictive framework for CPC behavior in real-world conditions.

Despite numerous efforts to describe conductive network formation in CPCs, existing models largely fail to capture the dynamic restructuring of percolation networks under mechanical deformation, especially the coupled effects of filler anisotropy, orientation, and Poisson-driven expansion. To address this gap, the present study aims to develop a dynamic statistical mechanics model that extends classical percolation theory by incorporating filler morphology, orientation distribution, and strain-induced network evolution. By integrating gain–loss kinetics and validating through Monte Carlo simulations, the proposed model quantitatively characterizes both the onset and progression of conductive networks under compressive strain, ultimately providing a predictive framework for designing strain-sensitive CPCs.

## 2. Materials and Methods

### 2.1. Statistical Mechanics Modeling

To develop a model that explains the conductive network formation of CPCs based on statistical mechanics, we extended the basic model of percolation theory to a more complex system, considering the shape, AR, and alignment of the conductive fillers. The following equation represents the conductive network connectivity probability using the Poisson distribution [42,43], based on the percolation theory as a function of the volume fraction of conductive fillers.

(1) $$P_{connect}=1-e^{-{\left(\phi -\phi_{c}\right)}^{\beta }}$$

(2) $$\phi =\frac{NV_{filler}}{V_{total}}$$

where $P_{connect}$ represents the probability of a conductive network in the CPCs, ϕ is the volume fraction of conductive fillers, $\phi_{c}$ is the critical volume fraction of percolation, and β is a sensitivity exponent related to percolation transition. *N* is the number of conductive filler particles, $V_{filler}$ is the volume of a single filler, and $V_{total}$ is the total volume of CPCs. This basic percolation model describes the sharp increase in connectivity probability that occurs when the volume fraction exceeds the critical threshold, marking the onset of a continuous conductive network within the composite.

However, since this equation is governed solely by the number and volume of fillers, it does not reflect morphological effects. Therefore, an extended model is required to account for the geometric characteristics of the fillers. Conductive fillers can typically be modeled as idealized shapes such as spheres, rods, or plates. For a given volume fraction and filler shape, a configuration consisting of smaller and more numerous particles provides a larger total accessible surface area for contact compared to fewer, larger particles. This increase in surface area can enhance the probability of inter-particle connectivity, which is critical for the formation of a conductive network. Therefore, considering the shape and contact area, the expanded equation is as follows:

(3) $$P_{connect}=1-e^{-{\left(\phi -\phi_{c}\right)}^{\beta }{\left(\frac{A_{f}}{V_{f}}\right)}^{\alpha }}$$

The surface area $A_{f}$, volume $V_{f}$, and their ratio $A_{f}/V_{f}$ for typical filler geometries are defined as

(4) $$V_{f}=\left\{\begin{array}{c} V_{sphere}=\frac{4\pi r^{3}}{3}\\ V_{rod}=\pi r^{2}l=AR\pi r^{3},\;AR=l/r\\ V_{plate}=wlt=\frac{w^{3}}{{AR}_{1}{AR}_{2}},\;{AR}_{1}=w/h,\;{AR}_{2}=w/t\; \end{array}\right.$$

(5) $$A_{f}=\left\{\begin{array}{c} A_{sphere}=4\pi r^{2}\\ A_{rod}=(2\pi r^{2}+2AR\pi r^{2})\\ A_{plate}=2w^{2}({\frac{1}{AR}}_{1}+{\frac{1}{AR}}_{2}+1/{AR}_{1}{AR}_{2}) \end{array}\right.$$

(6) $$A_{f}/V_{f}=\left\{\begin{array}{c} \frac{3}{r}\;(\mathrm{sphere})\\ \frac{2(1+AR)}{ARr}\;(\mathrm{rod})\\ \frac{2({AR}_{1}+{AR}_{2}+1)}{w}\;(\mathrm{plate}) \end{array}\right.$$

In these equations, α is a shape-sensitivity coefficient representing the effect of surface area on network formation. r denotes the radius of spheres and rods, l is the rod length, and w, h, and t are the width, height, and thickness of plate-like fillers. AR is defined as l/r for rods, and as w/h and w/t for plate fillers.

This formulation demonstrates that increasing the surface area-to-volume ratio enhances the probability of filler-to-filler contact, thereby improving network connectivity. For a fixed filler volume, smaller particles (with higher $A_{f}/V_{f}$) lead to higher connectivity probability. Furthermore, in the case of anisotropic fillers such as rods and plates, higher ARs increase the accessible contact area, thereby enhancing conductive network formation.

Asymmetric fillers such as rods and plates tend to align within the CPC matrix and, thus, an additional alignment term is introduced to account for this behavior. The resulting equation incorporating this term is given by

(7) $$P_{connect,0}=1-e^{-{\left(\phi -\phi_{c}\right)}^{\beta }{\left(\frac{A_{f}}{V_{f}}\right)}^{\alpha }D}\;\;$$

where D is the orientation diversity parameter, defined using orientational entropy as [44]

(8) $$D=\frac{H}{H_{max}}\;,\;\;H=-\sum_{i=1}^{n}p_{i}\log p_{i}\;$$

Here, $p_{i}$ denotes the fraction of particles falling within the i-th angular bin. The parameter H measures the orientational entropy, and D is normalized by its maximum value $H_{max}$. A higher D indicates a more isotropic filler orientation, while a lower value reflects stronger alignment along a specific direction. When fillers are uniformly distributed in all directions, D=0; conversely, when they are perfectly aligned, D→0 [45].

**Figure 1:**
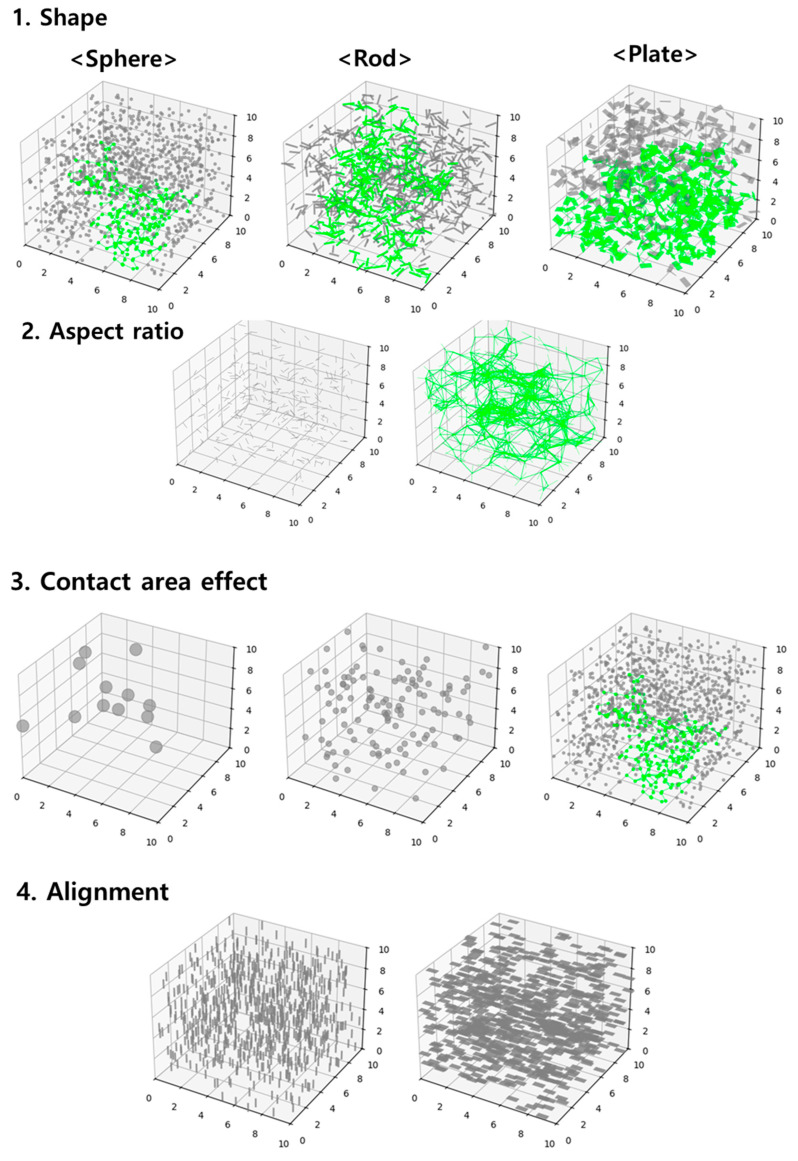
Visualization of conductive network formation and infinite clusters (green lines) in CPCs with various filler shapes and orientations.

The final form of the statistical mechanics model integrates the effects of filler shape, contact area, and alignment. This comprehensive formulation enables quantitative analysis of complex conductive network formation mechanisms in CPC systems. The influence of filler characteristics on connectivity probability is illustrated in Figure 1.

Figure 1 illustrates the formation of conductive networks in CPCs, where the green lines represent the percolated pathways (infinite clusters) and the fillers involved in these connections are also highlighted in green. Figure 1 demonstrates that infinite clusters are more easily formed in anisotropic fillers, such as rods and plates [37], due to their elongated geometries, which promote directional alignment and extended interfacial contact.

Additionally, it highlights that at an equal volume fraction, anisotropic fillers with higher ARs tend to exhibit higher connectivity probabilities compared to those with lower ARs. In the case of symmetric spherical fillers, an increase in the contact surface area enhances the probability of network formation, even when the volume fraction is constant. Conversely, when the alignment distribution is held constant, the connectivity probability is comparatively lower if the filler shape or surface characteristics are less favorable. These visual trends reinforce the effects of filler geometry, surface area, and alignment on the percolation behavior predicted by the model.

The Statistical mechanics model proposed in this study is an extension of classical percolation theory, which accounts for the geometric characteristics of conductive fillers. It estimates the probability of forming a conductive network based on three primary factors: the volume fraction of conductive fillers, the critical volume fraction at which percolation begins, and the surface-area-to-volume ratio of the fillers raised to a shape-sensitivity exponent. This formulation allows the model to reflect how different filler morphologies—such as spheres, rods, and platelets—influence the likelihood of interparticle contact and connectivity. Unlike the dynamic model, which considers strain-induced changes, the SM model assumes a fixed, undeformed filler configuration. It serves as a baseline to understand the effect of filler geometry on the onset of electrical connectivity. Monte Carlo simulation results under undeformed conditions confirm that anisotropic fillers with higher surface-to-volume ratios exhibit significantly lower percolation thresholds compared to isotropic ones, validating the model’s predictions.

### 2.2. Dynamic Statistical Mechancis Modeling

The statistical mechanics model of CPCs presented in Section 2.1 was developed based on percolation theory to describe the formation of conductive networks within the composite material under static conditions, accounting for the arrangement, shape, and alignment of internal conductive fillers. However, when CPCs are applied as sensors, the dynamic formation and evolution of the conductive network under mechanical deformation becomes increasingly significant. Therefore, it is essential to extend the model into a dynamic formulation that captures the behavior of the network under compressive strain.

To this end, we refer to the coagulation equation proposed by Marian Smoluchowski, which separates system evolution into gain and loss terms to describe the time-dependent aggregation of colloidal particles due to collisions. The Smoluchowski coagulation equation is expressed as follows [41]:

(9) $$\frac{dn_{k}(t)}{dt}=\frac{1}{2}\sum_{i+j=k}K\left(i,j\right)n_{i}\left(t\right)-n_{k}(t)\sum_{j=1}^{\infty }K(k,j)n_{j}\left(t\right)$$

In this equation, $n_{k}(t)$ represents the concentration of particles of size k at time t and $K\left(i,j\right)$ is the coagulation kernel that defines the probability at which particles of sizes i and j collide and merge to form a new particle of size k. The first term on the right-hand side describes the gain of particles of size k due to the coagulation of smaller particles. The second term represents the loss of particles of size k, as they combine with other particles to form larger aggregates.

**Figure 2:**
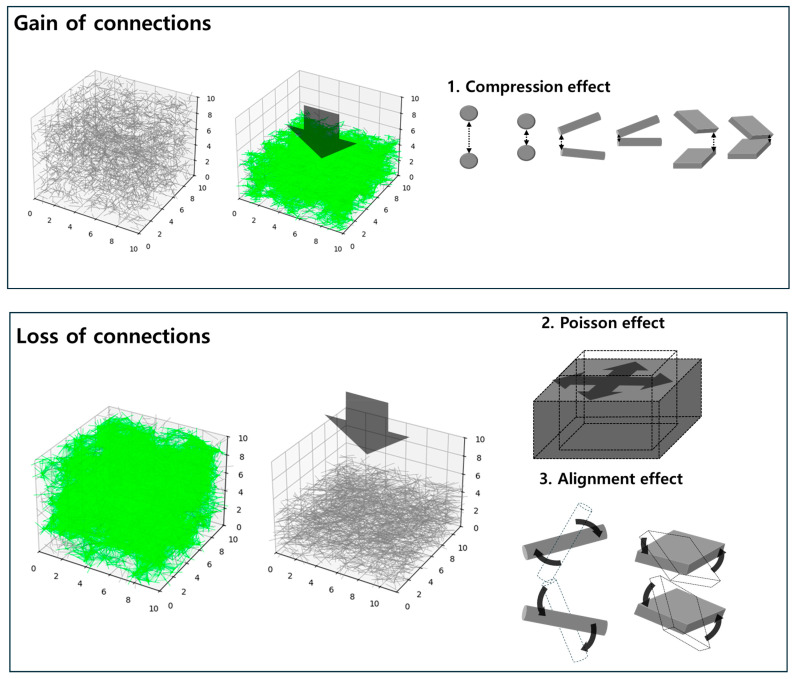
Schematic illustration of gain and loss mechanisms affecting conductive network formation (green lines) in CPCs under compressive strain.

Similarly, as illustrated in Figure 2, the compressive deformation of CPCs results in two competing phenomena. First, the reduction in inter-filler distance promotes the formation of new conductive pathways, thereby enhancing network connectivity. Second, deformation induces disconnection effects due to lateral expansion perpendicular to the compression axis primarily caused by the Poisson effect and the reorientation of fillers, which leads to increased alignment and reduced random contact opportunities. These opposing mechanisms collectively influence the dynamic evolution of the conductive network under compressive strain.

First, the compressive deformation of CPCs along the *Z*-axis due to applied pressure results in a reduction in overall volume, which can be expressed as

(10) $${V_{total}}^{\prime }=V_{total}\left(1-\varepsilon \right){\left(1+\nu \varepsilon \right)}^{2},\;(\varepsilon >0)$$

Here, ε denotes the compressive strain applied along the *Z*-axis and ν is the Possion’s ratio of the CPC material. The composite contracts along the compression axis while expanding laterally in the XY-plane due to the Poisson effect. When considering only the volumetric effect of compression, the volume fraction of conductive fillers increases as the total volume decreases. This strain-induced increase in filler volume fraction is defined as

(11) $$\phi_{gain}\left(\varepsilon \right)=\phi_{0}(\frac{1}{\left(1-\varepsilon \right)}-1)$$

where $\phi_{gain}\left(\varepsilon \right)$ is the effective increase in filler volume fraction due to compression and $\phi_{0}$ is the initial filler volume fraction before compression. The total gain term in the dynamic statistical mechanics model for CPCs under compressive strain is defined as

(12) $$\mathrm{Gain}\left(\varepsilon \right)={\left(\phi_{Gain}\left(\varepsilon \right)-\phi_{gain,c,\varepsilon }\right)}^{\beta^{\prime }}{\left(\frac{A_{f}}{V_{f}}\right)}^{\alpha^{\prime }}D_{0}$$

where this term is activated under the condition

(13) $$\left(\phi_{Gain}\left(\varepsilon \right)-\phi_{gain,c,\varepsilon }\right)=\left\{\begin{array}{c} {>0,{\mathrm{if}\phi }_{Gain}\left(\varepsilon \right)>\phi }_{gain,c,\varepsilon }\\ 0,\;otherwise \end{array}\right.$$

In this equation, $\mathrm{Gain}\left(\varepsilon \right)$ represents the increase in network connectivity driven by the densification of conductive fillers under compressive strain. ε denotes the critical volume fraction above which new conductive pathways begin to form. $\phi_{gain,c,\varepsilon }$ was identified as the minimum volume fraction at which percolation was initiated under compressive strain. This reflects the threshold-like behavior of percolation, where no significant gain in connectivity occurs until this critical point is surpassed. The exponent $\beta^{\prime }$ captures the strain-sensitive percolation response. A higher $\beta^{\prime }$ indicates a sharper increase in connectivity once the local filler density surpasses the critical threshold, reflecting a rapid percolation transition, while $\alpha^{\prime }$ characterizes the influence of contact area, quantified by the surface-to-volume ratio. A higher $\alpha^{\prime }$ indicates that increased contact area between fillers—especially in anisotropic or smaller particles—more effectively promotes conductive pathways, emphasizing the role of geometric surface exposure in percolation. $D_{0}$ denotes the initial orientational diversity of fillers, reflecting the baseline alignment distribution before deformation.

In contrast to the gain term, the loss term accounts for the reduction in network connectivity caused by lateral expansion in the XY-plane due to the Poisson effect, as well as the increase in both center-to-center and effective distances between fillers resulting from alignment. The volume fraction loss of conductive fillers induced by this effect is expressed as

(14) $$\phi_{Loss}\left(\varepsilon \right)=[\frac{{\left(1+\nu \varepsilon \right)}^{2}-1}{\phi_{0}}]$$

Here, $\phi_{Loss}\left(\varepsilon \right)$ represents the effective volume fraction loss arising from the increased separation between fillers due to transverse expansion. This term increases with both compressive strain and Poisson’s ratio, but decreases with higher initial filler volume fraction $\phi_{0}$. The equation captures the diluting effect of deformation in the XY-plane on filler proximity, which undermines conductive path formation. The alignment-induced loss component, which accounts for the rotation and reorientation of asymmetric fillers under compression, is defined as

(15) $$D\left(\varepsilon \right)=\left\{\begin{array}{c} 1,\;for\;spherical\;fillers\\ D_{0}\left[\frac{\left(1-\varepsilon \right)}{{\left(1+\nu \varepsilon \right)}^{2}}\right],\;\;\mathrm{>f}\mathrm{>o}\mathrm{>r}\;\mathrm{>r}\mathrm{>o}\mathrm{>d}\;\mathrm{>a}\mathrm{>n}\mathrm{>d}\;\mathrm{>p}\mathrm{>l}\mathrm{>a}\mathrm{>t}\mathrm{>e}\;\mathrm{>f}\mathrm{>i}\mathrm{>l}\mathrm{>l}\mathrm{>e}\mathrm{>r}\mathrm{>s}\; \end{array}\right.$$

Here, $D\left(\varepsilon \right)$ represents the orientational diversity, which decreases as asymmetric fillers (rods or plates) become increasingly aligned in the XY-plane under compressive strain. This alignment reduces the likelihood of random contact and thus increases connectivity loss.

The overall Loss term in the dynamic model combines both Poisson effect-induced volume loss and orientation-induced contact reduction, and is defined as

(16) $$\mathrm{Loss}\left(\varepsilon \right)={{(\phi }_{Loss}\left(\varepsilon \right)-{(\phi }_{loss,c,\varepsilon }/\phi_{0}))}^{\gamma }{D\left(\varepsilon \right)}^{\lambda }={\left[\frac{{\left(1+\nu \varepsilon \right)}^{2}-1}{\phi_{0}}\right]}^{\gamma }D_{0}{\left[\frac{\left(1-\varepsilon \right)}{{\left(1+\nu \varepsilon \right)}^{2}}\right]}^{\lambda }$$

This expression is applied under the condition

(17) $$\left({(\phi }_{Loss}\left(\varepsilon \right)-{(\phi }_{loss,c,\varepsilon }/\phi_{0})\right)=\left\{\begin{array}{c} >0,{\mathrm{if}\;\phi }_{Loss}\left(\varepsilon \right)>{(\phi }_{loss,c,\varepsilon }/\phi_{0})\\ 0,\;\;otherwise \end{array}\right.$$

Here, $\phi_{loss,c,\varepsilon }$ is the critical loss volume fraction under compression. Specially, $\phi_{loss,c,\varepsilon }$ corresponded to the volume fraction below which connectivity degraded under lateral expansion (due to Poisson’s ratio effects). The conditional form reflects the fact that disconnection of the network does not occur during the early stages of compression unless the loss-induced separation exceeds a threshold. Moreover, this critical condition becomes more stringent in systems with higher particle counts, as shown by the division with $\phi_{0}$. The exponents γ and λ, respectively, represent the sensitivity of connectivity loss to two competing strain-induced effects: the decrease in filler density due to lateral expansion (Poisson effect) and the increase in filler alignment under compression. A higher γ indicates that small changes in filler spacing significantly disrupt network connectivity, while a higher λ means that even modest orientation alignment can strongly reduce random inter-particle contacts, weakening the percolated structure.

Finally, the complete equation for the dynamic statistical mechanics model, which quantifies conductive network connectivity in CPCs under compressive strain, is expressed as

(18) $$P_{connect}\left(\varepsilon \right)=1-e^{-k[\mathrm{Gain}\left(\varepsilon \right)-\mathrm{Loss}\left(\varepsilon \right)]}$$

The total connectivity probability that combines both initial and strain-dependent components is given by

(19) $$P_{connect.total}=P_{connect,0}+{(1-P_{connect,0})P}_{connect}\left(\varepsilon \right)$$

Here, $P_{connect,0}$ is the initial connection probability from the static Statistical Mechanics Model before compression, and k is a scale factor that modulates the sensitivity of the dynamic model to strain. In this study, the statistical mechanics-based modeling approach, inspired by the Smoluchowski aggregation framework, was employed to dynamically describe the evolution of conductive network connectivity under compressive strain. The model captures the contributions of structural parameters such as filler distance, Poisson-induced expansion, and the orientation and alignment behavior of asymmetric conductive fillers. These effects were systematically separated into gain and loss terms, allowing for a comprehensive analysis of dynamic percolation in CPCs.

### 2.3. Monte Carlo Computer Simulation

Monte Carlo simulations were implemented using Python 3.10 on Google Colab, utilizing GPU acceleration via Google Compute Engine (L4 GPU). The CPC system was modeled as a 3D cubic domain with dimensions of 10 × 10 × 10, in which conductive fillers were randomly distributed without overlapping. Two fillers were considered to be electrically connected if the surface-to-surface distance between them was less than a critical threshold of 0.25.

A conductive network was defined as an infinite cluster when a continuous connection spanned from one plane to the opposite plane along the *Z*-axis. The connectivity probability was calculated as the ratio of the number of fillers participating in the infinite cluster to the total number of fillers in the system.

To ensure statistical robustness, each simulation was repeated at least 30 times under identical conditions. This repetition enabled the analysis of connectivity distribution and the validation of the proposed dynamic statistical mechanics model.

The experiments were conducted by controlling key variables such as the volume of a single filler, the number of fillers, the shape of the fillers, and the ARs of asymmetric fillers, specifically the ARs of rods and plates. The alignment distribution was set to random. The result is represented as the connectivity probability of the conductive network, which was calculated by dividing the number of fillers used in the infinite cluster connection by the total number of fillers.

**Figure 3:**
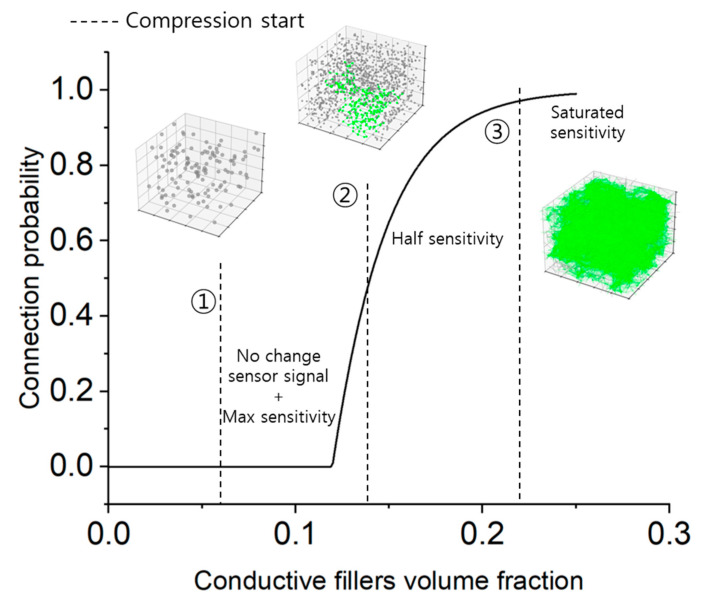
Schematic representation of conductive network evolution under compression for CPCs with different filler configurations.

To validate the dynamic statistical mechanics model, simulations were conducted to observe the evolution of conductive network connectivity in CPCs under compression. As shown in Figure 3, three different filler configurations—spherical, rod-shaped, and plate-shaped conductive fillers—were investigated.

In the first condition, where the initial volume fraction of conductive fillers is low, a conductive network does not initially form. However, the relative increase in connectivity during compression is maximal, resulting in the highest sensor sensitivity. In the second condition, the network is in the process of forming, so the connectivity increases at a moderate rate and the sensor sensitivity is approximately halved. In the third condition, the conductive network is already fully formed prior to compression, leading to minimal further connectivity and thus negligible sensitivity change. These results indicate that, theoretically, CPCs in the first condition—where the change in connectivity is most significant—are best suited for strain-sensitive applications.

It should be noted, however, that these findings are based on simulations assuming complete compression and a Poisson’s ratio of zero. To investigate the effects of realistic deformation behaviors, the model was extended to include Poisson’s ratios of 0.3 and 0.5. Additionally, the influence of asymmetric filler rotation and alignment was examined. The comparative results from these extended simulations were used to analyze the impact of lateral expansion and filler orientation on conductive network dynamics in CPCs.

## 3. Results

*Validation of Statistical Mechanics Model*

**Figure 4:**
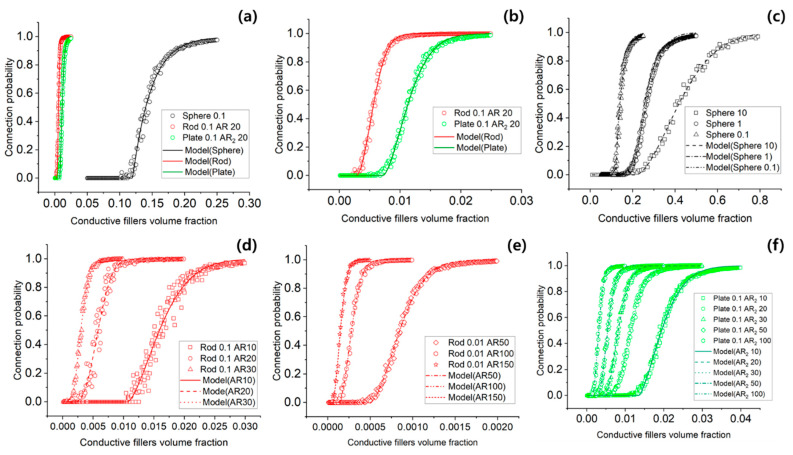
Comparison of Monte Carlo simulation results and the extended statistical mechanics model for connectivity probability and critical volume fraction in CPCs with varying conductive filler geometries. (**a**) conductive fillers 3 shapes(sphere, rod, and plate), volume 0.1 (**b**) conductive fillers 2 shapes(rod and plate), volume 0.1 (**c**) sphere shape, 3 volume 0.1, 1, and 10 (**d**) rod shape volume 0.1, AR 10, 20, and 30 (**e**) rod shape volume 0.01, AR 50, 100, and 150 (**f**) plate shape volume 0.1, AR_2_ 10, 20, 30, 50, and 100.

The results of the Monte Carlo simulations for the connectivity probability of the conductive network in conductive polymer composites (CPCs) are summarized in Figure 4 and Table 1.

Figure 4a,b present connectivity probability as a function of filler volume fraction for three types of conductive fillers: spherical, rod-shaped, and plate-shaped. When the filler volume was fixed at 0.1, the critical volume fraction required to form a percolated network was significantly lower for anisotropic fillers. Specifically, rod-shaped fillers exhibited a critical volume fraction of 0.00203, and plate-shaped fillers showed 0.00669, whereas spherical fillers required 0.11965. This indicates that anisotropic fillers can form conductive networks more efficiently.

Figure 4c investigates the role of contact area using spherical fillers by varying the individual filler volume while keeping the total volume fraction constant. As the size of individual spherical fillers increased, the total surface area decreased, resulting in higher critical volume fractions. This result indicates that contact area plays a significant role in network formation, which is not captured by traditional percolation models.

Figure 4d,e show the influence of the AR for rod-shaped fillers. With a constant filler volume of 0.1, increasing AR to 10, 20, and 30 led to monotonic decreases in the critical volume fraction to 0.01066, 0.00203, and 0.00203, respectively. When the filler volume was reduced to 0.001 and the AR was increased to 50, 100, and 150, the critical volume fraction further decreased to 0.0005, 0.00012, and 0.00005, respectively.

Figure 4f presents the results for plate-shaped fillers, modeled as rectangular particles. While the AR_1_ was fixed at 2, the AR_2_ varied from 10 to 100. As AR_2_ increased, the critical volume fractions decreased from 0.01344 to 0.00142, demonstrating a consistent trend of improved network formation with increasing geometric anisotropy.

Table 1 summarizes the optimized parameters of the extended statistical mechanics (SM) model across all filler shapes and conditions. The RMSE values across these simulations ranged from 0.0145 to 0.0449, corresponding to an average relative error of approximately 4.5%. These values are in line with the expected range for 3D percolation systems, where critical exponents typically fall between 1.3 and 2.4 [46,47,48].

**Figure 5:**
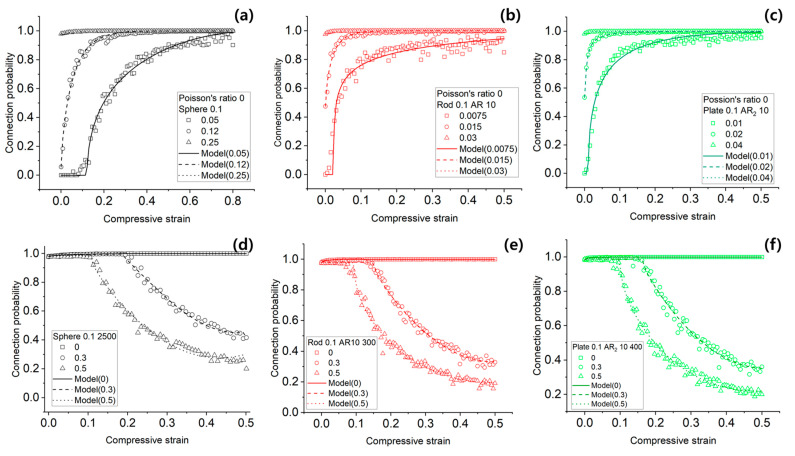
Comparison of Monte Carlo simulation and dynamic statistical mechanics model predictions of conductive network connectivity in CPCs under compressive strain. (**a**) sphere shape, initial particle volume fraction 0.05, 0.12, and 0.25 (**b**) rod shape, initial particle volume fraction 0.0075, 0.015, and 0.05 (**c**) plate shape, initial particle volume fraction 0.01, 0.02, and 0.04 (**d**) sphere shape, initial volume fraction 0.25, poisson’s ratio 0, 0.3, and 0.5 (**e**) rod shape, initial volume fraction 0.03, poisson’s ratio 0, 0.3, and 0.5 (**f**) plate shape, initial volume fraction 0.04, poisson’s ratio 0, 0.3, and 0.5.

Figure 5 presents validation results for the proposed dynamic statistical mechanics model under compressive strain. Figure 5a–c illustrate the evolution of network connectivity for spherical, rod-shaped, and plate-shaped fillers, respectively, under a fixed filler volume fraction of 0.1 and Poisson’s ratio of 0.

For spherical fillers in Figure 5a, three initial volume fractions were considered: 0.05, 0.12, and 0.25. For $\phi_{0}=0.05$, which is below the critical threshold, the network did not form until a compressive strain of approximately 0.1. Connectivity increased gradually and saturated at strain ≈ 0.8. For $\phi_{0}=0.12$, just above the threshold, percolation occurred immediately under compression and reached full connectivity at strain ≈ 0.4. In the case of $\phi_{0}=0.25$, connectivity was already saturated at zero strain, and further compression had little to no impact.

Figure 5b,c show similar trends for rod and plate-shaped fillers. At low initial volume fractions, the network forms progressively under compression. At moderate $\phi_{0}$, the increase in connectivity becomes limited. At high $\phi_{0}$, the network is already formed before compression, and no further gain is observed.

Table 2 reports the optimized gain term parameters for each filler and volume condition. RMSE values in this dataset ranged from 0.0004 to 0.0421, indicating strong model agreement with simulation results.

Figure 5d–f show simulation results under high Poisson’s ratios (ν = 0.3 and 0.5) for spherical, rod, and plate fillers. These cases highlight the impact of lateral expansion during compression. In Figure 5d, for spherical fillers at $\phi_{0}=0.25$, network connectivity declined as Poisson’s ratio increased. Similar or more severe reductions are shown in Figure 5e,f for rod and plate fillers, respectively.

Table 3 summarizes the optimized loss term parameters. RMSE values ranged from 0.0188 to 0.0253, demonstrating that the loss term effectively captures the degradation of connectivity under lateral deformation.

## 4. Discussion

The simulation results clearly demonstrate that anisotropic fillers, such as rods and plates, are significantly more effective at forming conductive networks than isotropic spherical fillers. This effectiveness is attributed to their elongated or flattened geometries, which allow for greater spatial reach and increased contact opportunities. As a result, the critical volume fraction required to initiate percolation is considerably lower for anisotropic fillers. These findings are consistent with experimental observations of carbon nanotubes (CNTs) and graphene-based fillers, which are known for their high aspect ratios and superior conductive performance.

Several prior experimental and computational studies support these observations. For instance, Chang et al. [36] reported a marked decrease in percolation threshold with increasing aspect ratio in curved fiber-reinforced composites, using both simulation and experimental validation. Similarly, Bilotti et al. [26] demonstrated that adding high-aspect ratio CNTs to a polymer matrix significantly enhanced conductivity and strain sensitivity due to improved network formation. Wu et al. [49] also confirmed through combined molecular dynamics simulations and experiments that the alignment of nanorods facilitates conductive pathway development. These studies validate the predictive capability of our model and underscore the practical relevance of anisotropic filler design in CPCs.

Furthermore, the influence of contact area, particularly for spherical fillers, is well captured by the extended statistical mechanics model. As shown in Figure 4c, increasing the size of individual fillers while maintaining the total volume fraction leads to a decrease in total surface area, raising the percolation threshold. This result highlights the importance of including surface area effects in connectivity models, which are typically neglected in classical percolation theory.

The aspect ratio-dependent results (Figure 4d–f) reinforce the importance of geometric anisotropy in reducing the percolation threshold. For both rods and plates, increasing AR leads to a sharper decline in $\phi_{c}$, indicating more efficient network formation. This trend continues even in high-AR cases such as AR = 150, which closely resembles the geometry of CNTs commonly used in real-world CPC applications.

Validation of the dynamic statistical mechanics model under compression (Figure 5) further supports its robustness. At zero Poisson’s ratio, only the gain term is active, allowing the model to accurately capture the progressive formation of conductive networks with increasing strain, particularly in systems near or below percolation threshold. These findings demonstrate that the model successfully replicates strain-induced densification effects in CPCs.

The effect of Poisson’s ratio becomes critical in more realistic systems, where lateral expansion reduces filler proximity. The loss term of the model, governed by the parameters, accounts for this behavior. Increased ν leads to greater inter-filler distances in the XY-plane and potential misalignment, especially in anisotropic fillers. As shown in Figure 5d–f, connectivity is increasingly lost in such systems, with rod and plate fillers showing more pronounced declines than spherical fillers due to their alignment sensitivity.

The strong agreement between model predictions and simulation data—evidenced by low RMSE values across all scenarios—demonstrates the model’s applicability to a wide range of geometries and loading conditions. The integration of both gain and loss mechanisms within a unified framework offers a comprehensive understanding of conductive network evolution in CPCs, particularly under mechanical deformation. These insights are essential for the rational design of flexible electronic and sensor materials, where precise control over conductivity and strain sensitivity is required.

The present model assumes idealized filler shapes and uniform dispersion, without considering possible filler agglomeration, interfacial debonding, or viscoelastic relaxation under cyclic loading. Furthermore, the current framework does not account for time-dependent effects such as creep and dynamic fatigue that may occur in real-world applications. To address these gaps, future work should aim to incorporate filler–filler and filler–matrix interaction mechanisms, viscoelastic matrix behavior, and experimental validation under various environmental and operational conditions. These improvements will further enhance the model’s predictive capability for practical use in advanced strain-sensitive CPC devices.

## 5. Conclusions

In this study, a statistical mechanics model and its extension to a dynamic statistical mechanics model was developed to systematically analyze the formation and evolution of conductive networks in conductive polymer composites (CPCs). The statistical mechanics model quantified percolation probability by incorporating key filler characteristics such as geometry (spheres, rods, and plates), aspect ratio, and surface-to-volume ratio. It successfully captured the reduction in percolation threshold associated with anisotropic fillers, as validated through Monte Carlo simulations.

Building on this foundation, a dynamic statistical mechanics model was proposed to describe the strain-dependent network behavior of CPCs under compressive deformation. Using Smoluchowski-type gain–loss formalism, the model accounts for conductive network growth (gain) resulting from filler densification and network degradation (loss) due to lateral expansion from the Poisson effect and filler alignment. The model effectively predicted the nonlinear evolution of connectivity across a range of Poisson ratios and filler morphologies, and its results demonstrated strong agreement with simulation data, as indicated by low RMSE values.

Overall, the proposed models provide a physically grounded, computationally efficient framework for understanding and predicting the conductive behavior of CPCs under mechanical deformation. These results demonstrate a coherent link between the mathematical framework established in this study and the research objectives defined at the end of the Introduction, showing that the developed dynamic statistical mechanics model successfully captures the key physical factors—filler morphology, aspect ratio, and strain-dependent network evolution—and provides quantitative predictions that are consistent with Monte Carlo simulation results. This correlation confirms the model’s practical relevance for designing strain-sensitive CPC-based sensors as intended. They are particularly well suited for optimizing strain-sensitive devices such as flexible pressure sensors used in wearable electronics. Future work will focus on expanding the model to include viscoelastic behavior, filler–filler aggregation, and interfacial debonding, thereby enhancing its applicability to complex, real-world operational conditions.

## Figures and Tables

**Table 1:** Optimized parameters of the extended statistical mechanics model for CPCs.

$\mathrm{Shape}/V_{f}/$AR (AR_2_)	β	α	$\phi_{c}$	RMSE
Sphere/0.1	1.02	1.54	0.11965	0.0308
Sphere/1	1.6	2.33	0.18539	0.0394
Sphere/10	1.73	2.96	0.2148	0.021
Rod/0.1/10	1.55	2.78	0.01066	0.0449
Rod/0.1/20	2.41	4.39	0.00203	0.0302
Rod/0.1/30	1.86	3.7	0.00126	0.0163
Rod/0.01/50	1.73	3.33	0.0005	0.0177
Rod/0.01/100	1.95	3.93	0.00012	0.0191
Rod/0.01/150	1.86	3.84	0.00005	0.0192
Plate/0.1/10	1.5	1.46	0.01344	0.0194
Plate/0.1/20	1.8	1.59	0.00669	0.0188
Plate/0.1/30	1.71	1.48	0.00479	0.0145
Plate/0.1/50	1.86	1.54	0.00283	0.0149
Plate/0.1/100	1.85	1.46	0.00142	0.0179

**Table 2:** The optimized gain term parameters of the dynamic statistical mechanics model of CPCs at Poisson’s ratio 0 and volume of filler 0.1.

$\mathrm{Shape}/\phi_{0}$/AR(AR2)	$\beta^{\prime }$	$\alpha^{\prime }$	$\phi_{gain,c,\varepsilon }$	RMSE
Sphere/0.05	0.51	0.003	0.00738	0.0346
Sphere/0.12	0.768	1.566	0	0.0188
Sphere/0.25	0.828	1.461	0	0.0004
Rod/0.0075/10	0.321	0.712	0.00015	0.0421
Rod/0.015/10	0.38	1.361	0.00014	0.0127
Rod/0.03/10	0.494	1.467	0.00008	0.0004
Rod/0.01/10	0.515	1.362	0.0001	0.0346
Rod/0.02/10	0.447	1.356	0.00006	0.0035
Rod/0.04/10	0.371	1.246	0.00016	0.0002

**Table 3:** Optimized loss term parameters of the dynamic statistical mechanics model for CPCs with a filler volume fraction of 0.1.

$\mathrm{Shape}/\phi_{0}$/AR(AR2)	ν	γ	λ	$\phi_{loss,c,\varepsilon }$	RMSE
Sphere/0.25	0	-	-	-	0.0004
	0.3	0.294	-	0.11636	0.0188
	0.5	0.248	-	0.10914	0.0253
Rod/0.03/10	0	-	-	-	0.0421
	0.3	0.276	0.326	0.09246	0.0215
	0.5	0.161	0.155	0.09701	0.025
Rod/0.04/10	0	-	-	-	0.0002
	0.3	0.251	0.294	0.10143	0.0238
	0.5	0.154	0.157	0.10285	0.024

## Data Availability

The original contributions presented in this study are included in the article. Further inquiries can be directed to the corresponding author.

## Abbreviations

The following abbreviations are used in this manuscript:

CPC	Conductive Polymer Composite
AR	Aspect ratio
